# Adaptation of Laser Microdissection Technique for the Study of a Spontaneous Metastatic Mammary Carcinoma Mouse Model by NanoString Technologies

**DOI:** 10.1371/journal.pone.0153270

**Published:** 2016-04-14

**Authors:** Nadia P. Castro, Anand S. Merchant, Karen L. Saylor, Miriam R. Anver, David S. Salomon, Yelena G. Golubeva

**Affiliations:** 1 Tumor Growth Factor Section, Mouse Cancer Genetics Program, National Cancer Institute, Frederick, MD, 21702, United States of America; 2 CCRIFX Bioinformatics Core, National Cancer Institute, Bethesda, MD, 20892, United States of America; 3 Pathology-Histotechnology Laboratory, Leidos Biomedical Research, Inc., Frederick National Laboratory for Cancer Research, Frederick, MD, 21702, United States of America; University of Torino, ITALY

## Abstract

Laser capture microdissection (LCM) of tissue is an established tool in medical research for collection of distinguished cell populations under direct microscopic visualization for molecular analysis. LCM samples have been successfully analyzed in a number of genomic and proteomic downstream molecular applications. However, LCM sample collection and preparation procedure has to be adapted to each downstream analysis platform. In this present manuscript we describe in detail the adaptation of LCM methodology for the collection and preparation of fresh frozen samples for NanoString analysis based on a study of a model of mouse mammary gland carcinoma and its lung metastasis. Our adaptation of LCM sample preparation and workflow to the requirements of the NanoString platform allowed acquiring samples with high RNA quality. The NanoString analysis of such samples provided sensitive detection of genes of interest and their associated molecular pathways. NanoString is a reliable gene expression analysis platform that can be effectively coupled with LCM.

## Introduction

The molecular analysis of heterogeneous populations of cells lacks the ability to distinguish between subtle changes in the molecular signature of normal and diseased tissue and the correlation of cellular molecular signatures with specific cell populations. LCM has proven to be a critical research tool facilitating the discovery of genes responsible in the disease onset and progression by isolating homogeneous cell subpopulation from complex tissues [1–10]. The majority of microdissection-based mRNA expression studies have been performed on frozen samples, using traditional protocols for target collection, LCM slide and lysis preparation [4, 6, 7, 11]. However, these procedures are not directly applicable to the input sample requirements of downstream targets assessment using NanoString nCounter gene expression analysis which doesn’t require RNA extraction and amplification, and uses a relatively large but still feasible input amounts of RNA in the analysis in terms of LCM [12].

We have chosen to conduct our study on a clinically relevant mouse model, which phenotypically and at the gene-expression level resembles the human triple-negative breast cancer (TNBC) molecular subtype and which metastasizes spontaneously to the lungs [13]. To evaluate the reliability of NanoString samples obtained with our LCM workflow for gene expression profiling, we conducted a comparison of gene expression for a set of genes in NanoString LCM samples versus in whole tissue samples that were analyzed by Affymetrix microarray. The present manuscript describes the adaptation of LCM methodology for collection of samples suitable for NanoString analysis, which allows generating high quality RNA lysates from homogenous cell populations opposed to heterogeneous whole tissue samples. The NanoString technology with the use of LCM focuses on highly defined histological areas to clarify discrete molecular changes in gene expression at a greater level of resolution.

## Materials and Methods

### Cell line and culture conditions

The murine mammary carcinoma cells line JygMC(A)-GFP/Luc [13] containing a dual reporter construct (GFP/Luciferase) was maintained in Dulbecco’s-minimal essential media (DMEM; Invitrogen), supplemented with 10% Fetal Bovine Serum (FBS). Cells were grown in medium containing 100 units/ml penicillin, and 100 μg/ml streptomycin at 37°C in 5% CO2.

### Mouse strain and animal care

Animals used in this study were female Balb/c athymic nude mice that were 8 weeks of age (National Cancer Institute). Animal procedures were conducted under conditions approved by the Frederick National Laboratory for Cancer Research, an AAALAC accredited institution that follows the Public Health Service Policy for the Care and Use of Laboratory Animals outlined in the "Guide for Care and Use of Laboratory Animals" [14]; Frederick National Laboratory for Cancer Research ACUC 11–067 approval on 03/16/2012.

Animal diet consisted of Purina 5L79 pellets. Mouse cages were changed weekly, with additional cleaning and enrichment performed as needed. Animals were observed twice a day for the following features: coat condition, mobility (posture and movements), breathing, skin color, general alertness and responsiveness, tumor size and body weight. Maximum allowed tumor size was two centimeters in diameter, and maximum allowed weight loss was 20% of initial total body weight. Analgesia was performed by subcutaneous use of Marcaine (0.25%/0.1ml) and euthanasia by exposure to compressed CO_2_ gas at fill rate of 20% of the chamber volume per minute with one animal per chamber in its home cage. After ten minutes of exposure animals were observed for signs of unconsciousness (lack of respiration and pedal reflex, faded eye color) and subjected to bilateral thoracotomy.

### Mammary fat pad spontaneous metastasis model

*In vivo* experiments were performed as previously reported [13]. In brief, animals were anesthetized with isofluorane/O_2_ (to effect) and injected bilaterally into the fourth mammary fat pad with 20μl of 50,000 JygMC(A)-GFP/Luc cells/gland. Tumor growth was measured twice a week and monitored weekly by bioluminescent imaging after injection of luciferin. At 20–30 days after cell injection, the primary tumors were removed as a parallel of clinical settings.

### Samples

Normal mammary glands and normal lung parenchyma of 3 Balb/c athymic nude mice 6–8 weeks of age, and one or two fragments of primary mammary carcinoma and lung metastasis from 8 different mice were collected and embedded in Tissue-Tek® OCT Compound (OCT) (Sakura Finetek USA, Inc., Torrance, CA, USA) in a mixture of dry ice and 2-methylbutane (Fisher Scientific, Fair Lawn, NJ, USA) (S1 Fig). Frozen blocks were stored at -70°C prior to cryotomy and handled on dry ice at all times. The RNA quality control was performed for all collected material used for LCM. The statistical significance of differences in RNA integrity numbers was evaluated by two-tailed Student’s t-test with 95% confidence (p≤0.05).

### Cryotomy

We used a Leica CM 3050S (Leica Microsystems, GmbH, Nussloch, Germany) cryostat for obtaining serial 10μm tissue sections. At the beginning of serial sectioning three sections from each cryoblock were placed in 1.5 ml nuclease-free micro-centrifuge tube for high G-force (VWR, West Chester, PA, U.S.A.) and kept on dry ice for subsequent tissue RNA quality control. Every 6^th^ consecutive section was mounted onto a charged slide (Thermo Fisher Scientific, Waltham, MA, USA), stained with hematoxylin and eosin (H&E) and used as an LCM reference slide. Sequential serial sections were mounted onto metal frame PET membrane slides (MMI Molecular Machines & Industries, Glattbrugg, Switzerland) for laser cutting (S2 Fig). The number of sections mounted in the “window” area of the slide was determined by the size of the tissue. During preparation of LCM slides RNAse-free conditions were maintained throughout the procedure, as described previously [11], by using RNaseAWAY™ (Molecular BioProducts, San Diego, CA, USA) to wipe all the surfaces and tools, using 100% ethanol (AAPER Alcohol and Chemical Co., Shelbyville, KY, USA) to wipe the cryostat chamber, changing a blade for each block, and cutting away 15–20 μm of tissue from the face of a previously cut block. The “window” side of PET slides was labeled with solvent resistant adhesive label, and slides were exposed to ultraviolet light at 352 nm for 30 minutes before cryotomy for RNase-free conditions and better adherence of frozen sections to the membrane. RNAse-free slides were prechilled in the cryostat chamber for 2–5 minutes before section mounting. RNAse-free MMI SupportSlide (MMI Molecular Machines & Industries) was used to facilitate mounting of OCT sections onto PET slides (S2A–S2C Fig). Mounted LCM slides were kept at -70°C for two weeks prior to microdissection and handled on dry ice at all times.

### Pathology review and LCM documentation

**Fig 1:**
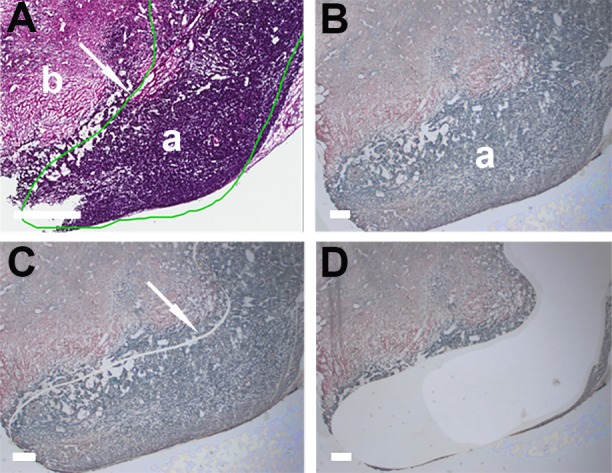
Pathology annotations of target area and documentation of carcinoma dissection by laser cutting. (A) Target area, carcinoma (a), was annotated by study pathologist on a digital image of reference H&E section of mammary gland (b) by green line (arrow); (B) View of LCM section with target area (a) on the dissecting screen of MMI CellCut microdissection instrument; (C) Dissecting screen view of the carcinoma area with the laser cut path (arrow); (D) Dissecting screen view of carcinoma area after retrieval of target cutout. A: Scale bar corresponds to 650 μm; B-D: Scale bars correspond to 300 μm.

Pathology evaluation of collected material was conducted by the study pathologist on the reference H&E slides (Fig 1A) for the presence of mammary carcinoma in tumors and metastasis in lungs, and absence of lesions in normal mammary gland and lung tissue.

**Fig 2:**
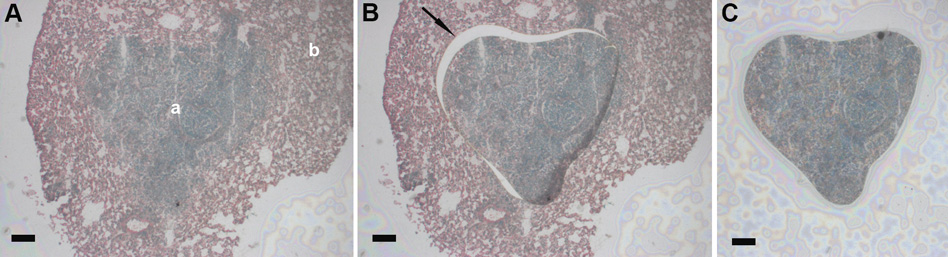
Documentation of lung metastasis removal from a mouse lung parenchyma by laser cutting. (A) Dissecting screen view of metastasis (a) in a mouse lung parenchyma (b); (B) Dissecting screen view of lung metastasis with the laser cut path (arrow); (C) View of LCM target cutout on the collection cap. Scale bars correspond to 100 μm.

Prepared H&E slides were digitally imaged with an Aperio Scan Scope®XT scanner according to the manufacturer’s directions, and quality of staining and morphological details was evaluated with Image Scope™ software. Digital images annotated by the study pathologist were used as reference slides during LCM of annotated targets for molecular analysis. The following LCM documentation was recorded for each sample: target area prior to LCM (Figs 1B and 2A), target area after laser cutting (Figs 1C and 2B), LCM area after target removal (Fig 1D) or views of LCM target on the collection cap (Fig 2C).

### Preparation of LCM slides

Fixation and staining of LCM slides was conducted under RNAse-free conditions in 50 ml conical polypropylene tubes (Falcon, Franklin Lakes, NJ, USA) containing 45 ml of solution, as previously described [11]. ProtectRNA™RNAse inhibitor (1:500) (Sigma, Saint Louis, MO, USA) was incorporated in the LCM staining protocol (Table 1, S3 Fig) to protect RNA from degradation during water containing steps performed at room temperature (RT) [15].

**Table 1:** LCM Staining Protocol.

Staining protocol step	Reagents	Duration	Temperature	Reference Image
Fixation	100% ethanol¹ with 3% acetic acid²	30 sec	-20°C	S3 Fig, A
OCT removal I	MethylGreen^3^ (1000μl) with Protect RNA^4^ (4 μl)	20 sec	RT	S3 Fig, B (1), C, E
OCT removal II	MethylGreen (1000μl) with Protect RNA (4 μl)	20 sec	RT	S3 Fig, B (1), C, E
Rinse	100% ethanol	10 sec	RT	S3 Fig, B(2), G
Stain	Cresyl Violet Acetate^5^/eosin^6^ mix (300μl)	30 sec	RT	S3 Fig, B (3), D, F
Dehydration I	100% ethanol	30 sec	RT	S3 Fig, B(4), G
Dehydration II	100% ethanol	30 sec	RT	S3 Fig, B(5), G
Clearing I	Xylene^7^	2 min	RT	S3 Fig, H
Clearing II	Xylene	3 min	RT	S3 Fig, H
Drying I	Air dry in the hood	5 min	RT	S3 Fig, I
Drying II	Desiccator	1–4 hour	RT	S3 Fig, J

^1, 2^ Mallinckrodf Baker Inc., Phillipsburg, NJ, USA

^3^ Vector Laboratories, Inc., Burlingame, CA, USA

^4, 5^ Sigma, Saint Louis, MO, USA

^6^ VWR International, Rednor, PA, USA

^7^ EMD Chemicals, Inc. Cincinnati, OH

LCM slides were moved on dry ice from a slide box into fixative that was prechilled on dry ice for 1 hour (fixative reached a temperature of -20°C). In the OCT removal step (Table 1), the solution was applied to sections twice, and the slides were drained on Kimwipes between applications. “One-step Cresyl Violet Acetate / Eosin Y” stain [11], was modified as follows: 75 μl of cresyl violet stock solution, 25 μl of eosin Y, 250 μl of RNAse-free water and 250 μl of 100% ethanol.

### Laser microdissection and preparation of lysates for NanoString analysis

Dissections of serial LCM slides were performed on a MMI CellCut Plus (MMI Molecular Machines & Industries, Glattbrugg, Switzerland) as previously described [15]. The dissection time was kept under 30 minutes. In order to fit into the dissection time frame, the drawings of target areas larger than 0.5 mm in diameter were done with 4x objective following by laser cutting of annotated targets with 10x objective under the following laser parameters: laser speed at 37%, laser focus at 78% and laser power at 41%. Large cutout targets were collected from the slides with Inox #5 forceps (Roboz Surgical Instrument, Co., Dumont, Switzerland), and small targets were overlapped on a cap for easy detachment as previously described [15]. Collected targets were placed into a 250μl nuclease-free PCR tube kept in wet ice and containing 5 μl of buffer RLT (Qiagen GmbH, Hilden, Germany), prepared according to the manufacturer’s instructions, and 0.3 μl of RNAseOut™ Rnase inhibitor (Invitrogen). The tube with targets from the first dissection session was then vortexed for 15 seconds, briefly centrifuged at maximum setting and returned on ice, making sure that the targets were completely submerged in lysis buffer. The targets from consecutive sessions were added to the tube upon the same protocol. After the completion of LCM the tubes were incubated at RT for 20 minutes. The targets were removed from the lysate as previously described [15], and the tubes were placed in dry ice. The samples were stored at -70°C for 4 weeks before NanoString analysis.

### Determination of sample quality and LCM workflow suitability for NanoString analysis

For quality control of tissue samples, lysis of collected frozen sections and RNA purification were performed with AllPrep® DNA/RNA Micro Kit (Qiagen GmbH, Hilden, Germany). Lysis buffer (350 μl) was added to each of the tubes and placed in dry ice, and then the tubes were vortexed for 2 minutes at maximum setting. After five-minute incubation at RT, tubes were vortexed for 30 seconds, and total RNA was extracted according to the manufacturer’s instructions. RNA quality control was performed on Agilent RNA 6000 PicoChip (Agilent Technologies, Santa Clara, CA). Quality assessment of the majority of RNA preparations was carried out in duplicates. Sample suitability for NanoString analysis was based on Agilent Bioanalyzer RNA Integrity Number (RIN) and an electropherogramm of RNA [16].

To evaluate the LCM workflow and the number of sections required for collection of 100 ng of RNA, we conducted a pilot study for RNA yield and quality in normal tissues and lesions (1 and 2 samples, respectively). The pilot analysis was designed to answer the following questions: 1) Does LCM slide preparation, dissection approach and NanoString lysis procedure preserve RNA in LCM targets? 2) What is RNA content per mm² of target area in lesions and normal tissue? 3) What tissue area (number of sections) will be required for acquisition of 100 ng of RNA per specific sample?

### NanoString analysis of LCM samples

Total RNA lysates were prepared for nCounter analysis [17]. Based on published literature, 103 mRNA genes and controls classified as embryonic stem cell (ESC), epithelial-mesenchymal transition (EMT) and mesenchymal-epithelial (MET) markers were selected for nCounter® Custom Gene Expression Assay (NanoStrings Technologies) [13]. The selected gene set including housekeeping genes used for normalization is listed in S1 Table. Assays were performed according to the manufacturer’s instructions (NanoStrings Technologies, Seattle, WA). The total RNA data have been deposited in NCBI’s Gene Expression Omnibus (GEO) and accessible through GEO Series accession number GSE63627.

### Quality control and data normalization of NanoString LCM samples

The data normalization was performed using the nSolver Analysis Software version 1.1 (NanoString Technologies, Seattle, WA) followed by background subtraction. The quality control (QC) parameters are based on the value of FOV (fields of view per sample) counts indicating imaging performance, binding density (a measure of sample saturation), positive and negative hybridization controls. A sensitivity level of 600 FOV counts was used for the analysis. The range for binding density should be between 0.05 and 2.25 across the samples. Positive control values should show linearity with corresponding dilutions and the negative control values should be low in a range from 0 to 10. Following the technical standardization, normalization was performed prior to analysis. Average of genes that are not expected to vary between samples (reference gene set) was used for normalization (*Hprt* NM_013556.2, *Oaz1* NM_008753.4, *Rpl27* NM_011289.3, *actin beta* NM_007393.1 and *Gapdh* NM_008084.1). The calculations for the background subtraction, correction factor, and normalization were performed as per standard NanoString analysis protocols [12].

### Affymetrix microarray of whole tissue samples

For global gene expression analysis (microarray), all RNA tissue samples were processed as previously reported [13]. Following extraction, RNA quality was accessed using the Agilent Bioanalyzer RNA Integrity Number (RIN) and RNA electropherogramm according to the manufacturer’s recommendations (Invitrogen, Carlsbad, CA). Genome-wide Affymetrix microarray hybridization and Microarray data processing also was performed as previously reported [13]. Microarray data have been deposited in NCBI’s Gene Expression Omnibus (GEO) and accessible through GEO Series accession number GSE63951.

### Statistical analysis of NanoString and microarray data

The raw data for microarray and NanoString study were pre-processed and normalized within Partek Genome Studio 6.6 (PGS, Version 6.6, Partek, Inc.) and nSolver v2.0.7.0, respectively. The custom set of 96 NanoString genes was extracted from the microarray dataset.

The data were then log-transformed and averaged across replicates for a particular sample group. Pearson’s Correlation statistic (p-value < 0.001) was then applied to test the significance of correlation for the expression profile of these 96 genes between the sample groups that were common across both the Microarray and NanoString analysis. A correlation heatmap was generated using Partek Genome Studio v6.6.

## Results and Discussion

### Determination of sample quality and LCM workflow suitability for NanoString analysis

**Fig 3:**
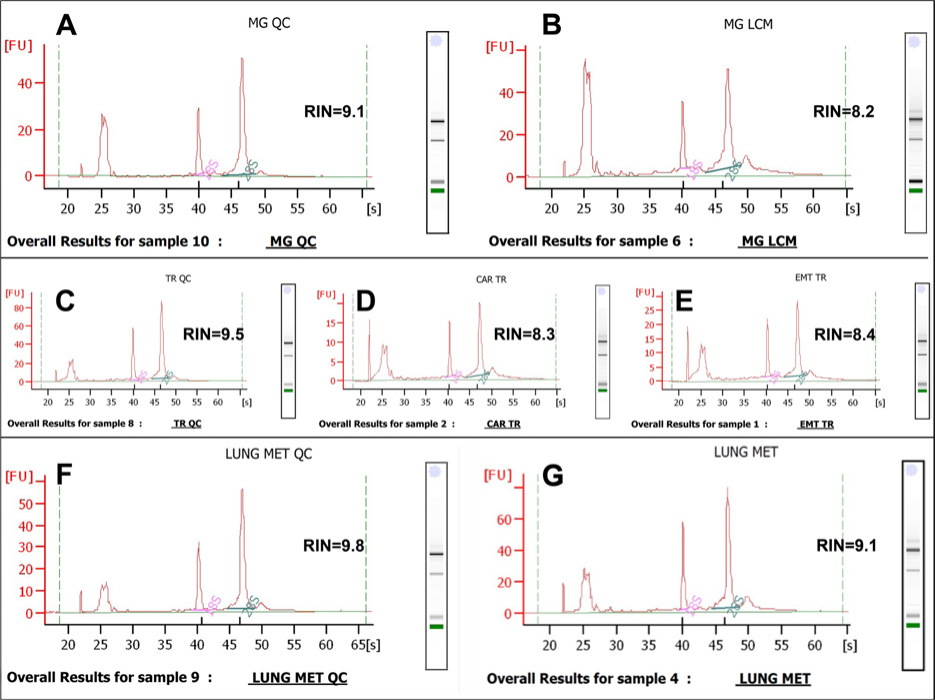
Representative Agilent electropherograms of high quality RNA retrieved from the control sections and corresponding LCM targets. (A, C, F) Frozen section of normal mammary gland (A), primary mammary tumor (C) and lung metastasis (F) placed directly in lysis buffer for RNA extraction. (B) LCM sample of normal mammary tissue; (D, E) LCM cell populations of primary mammary tumor: carcinoma (D) and EMT (E); (G) LCM sample of lung metastasis.

The NanoString platform allows some flexibility on RNA integrity of analyzed samples. For valid comparison 50% or more transcripts in the samples should be larger than 300 nt [12], which roughly corresponds to RIN number 5. Quality control of RNA in frozen tissue blocks embedded in OCT revealed high molecular integrity in acquired tissues with range of RIN numbers between 7.6 and 9.8 (Table 2, Fig 3), which is ideal for laser microdissection technique where some degradation of RNA occurs during LCM slide preparation and dissection, thus initial high integrity of tissue RNA is crucial.

**Table 2:** Evaluation of Tissue Integrity, LCM Workflow Suitability, Quality and Yield of RNA in LCM Targets.

Sample	Time in desiccator (hours)	Total collectedtissue area (mm²)	Total RNA per mm² of tissue (ng)	Approximated area required for collection of 100ng total RNA (mm²)	RNA Integrity number (RIN) Mean ±SD (n = 2)
Tumor 1 block, mixed targets, no LCM	0	40 (one frozen section)	37	2.7	9.6 ± 0.1
Tumor 2 block, mixed targets, no LCM	0	48 (one frozen section)	28	3.4	9.8 ± 0.1
Mammary gland block, mixed targets, no LCM	0	56.3 (one frozen section)	17	5.7	9.7 ± 0.2
Lung block, mixed targets, no LCM	0	90.6 (one frozen section)	5	21.7	9.1 ± 0.3
Metastatic lung block, mixed targets, as described in method section, no LCM	0	140 (one frozen section)	8	12.2	9.8 ± 0.2
Carcinoma 1, LCM	3.5	4	39	2.5	8.4 ± 0.1
EMT 1, LCM	3	7.4	30	3	8.5 ± 0.1
Carcinoma 2, LCM	5	6.8	54	2	8.5 ± 0.1
Mammary gland, LCM	4.5	27.4	18	5	8.3 ± 0.1
Lung parenchyma, LCM	4	63.7	7	14	7.6 ± 0.4
Lung metastasis, LCM	4	20.5	38	2.5	9.1 ± 0.2

The control of RNA quality of the starting material is mandatory not only because the high quality samples guarantee the validity of downstream results [18–20], but also for proper choice of downstream analysis applicable to the degree of RNA degradation in the tissue blocks, especially in case of unique samples. The analysis of intact RNA samples is not limited by the specific downstream application; the results obtained with one application can be verified with another downstream platform. The possibility of a failed LCM sample is also a consideration.

To preserve the RNA integrity and enhance nuclear visualization, we used water–based Methyl Green stain in OCT removal step instead of water. Besides being less detrimental to RNA than water [21–23], this stain followed by cresyl/eosin mixture also enhances nuclear visualization on LCM dissecting screen. The duration of cresyl/eosin staining step depends on the aging time of cresyl violet acetate stock solution; 1–1.5 year old stock provides good staining with 5 seconds of stain application. It is important to follow the proper procedure of tissue freezing in OCT media to avoid freezing artifacts which compromise tissue morphology and often prevent the acquisition of a good quality cryosection. The cryosectioning with anti-roll plate and a cryostat-automated function is preferable, since a flat section with even thickness better adheres to the membrane and facilitates uniform staining.

To evaluate the LCM workflow and the number of sections required for collection of 100 ng of RNA we conducted a pilot study for RNA yield and quality. Following the described above approach to NanoString sample preparation we acquired 5μl lysates from pilot tissues, adjusted their volumes to 350μl with buffer RLT, then extracted RNA and qualified it as described above. We calculated the required number of slides using the previously described approach [15]. Our LCM slide preparation, dissection and NanoString lysate preparation protocols effectively preserved RNA in the LCM targets (Table 2).

For a more efficient target collection we used a laser cutting technology with metal framed membrane slides which can accommodate multiple sections allowing complete pick-up of the dissected tissue from the slide [15, 24], contrary to laser capture with often incomplete pick-up and the necessity of target clean-up from contamination with non-specific tissue [4, 11]. Though, a clean and complete pick-up of laser-captured targets can be facilitated by use of CryoJane slides [25], a lysis procedure is labor intensive due to the low volume of the NanoString input. Since 5μl of lysate is not enough to lyse targets on the cap, the film with embedded targets should be peeled off the collection cap and cut into pieces for complete immersion in lysis buffer [25]. However, if the targets are smaller than 30–50μm in diameter, infrared laser capture is warranted, since UV laser damages RNA in small targets.

Storage of stained slides in the desiccator for up to 5 hours before LCM, and accumulation of dissected targets for 2.5 hours in lysis buffer on wet ice did not cause degradation of RNA neither in lesions, nor in normal tissue samples (Table 2). Flexibility with slide storage and lysis time is important for large-scale projects. When the dissected annotated target area was larger than required for 100ng of RNA, then before sample incubation at RT the default volume of lysis buffer (5μl) was increased proportionally to the size of the dissected area calculated by MMICellCut Software (S2 Table). Since the software features different drawing groups, the annotation of the target intended for the estimate of the total dissected area, and the annotation intended for actual dissection by the laser should be allocated to the different drawing groups. This approach allows distancing the laser path from the targets, when possible, to avoid the damaging effect of UV- laser on the integrity of the targeted cell population. The decrease of lysate volume (below 4 μl) during removal of cutout membranes from the tube can be adjusted with buffer RLT. We prefer to collect more material, adding on average two extra sections to the estimated number, because it is easier to work with volumes larger than 5μl during lysis and following removal of cutout membranes from the tubes. For pilot study, the samples with largest targets should be selected from the sample set, since enough LCM material should be collected and extracted with the minimal elution volume recommended by the manufacturer in order to measure RNA concentration by Nanodrop spectrophotometer. Measurements starting from 20ng per microliter can be reliably used for estimation of RNA content in LCM targets of the same sample set. Unfortunately, it is impossible to obtain uniform (by RNA concentration) LCM samples due to variable performance of column based RNA extraction kits; the average of three technical replicates of RNA content per mm² can differ by 30–40%. However, such RNA amount range in a sample set is accounted by the normalization algorithm of NanoString platform with high degree of correlation across a range of input amounts [12]. For all LCM samples used in this study and their respective information regarding dissected area, volume and LCM duration, see S2 Table.

### NanoString platform quality control of LCM samples

The FOV value across the sample set was close to the accepted sensitivity level of 600. Binding density was in the range of 0.05–2.25. Positive control values were linear to the dilutions and negative control values were in the range of 0 to 10. All LCM samples satisfied imaging quality control metrics for nCounter data analysis (S3 Table, S4 Fig).

### Quality of whole tissue samples for microarray analysis

Collected whole tissue samples were of high RNA integrity. RNA with RIN from 8.3 to 10 was used for microarray hybridization (S5 Fig).

### NanoString of LCM samples versus microarray of whole tissue samples

In this study we compared non-amplified LCM samples using a customized nCounter gene expression profile from NanoString technology with amplified whole tissue using a global Microarray profile. We observed high degree of confidence and concordance when comparing primary tumor (PT), lung metastasis (LungMets), normal mammary gland (NMG) and normal lung parenchyma (NL) (S6 Fig). The Pearson correlation scores for individual sample groups between NanoString and Microarray assays were very similar (Table 3).

**Table 3:** Pearson Correlation Scores for Individual Sample Groups Between NanoString and Microarray (p-value < 0.001).

Sample Group	Pearson Score
Primary Tumor	0.80
Lung Metastasis	0.81
Normal Mammary Gland	0.79
Normal Lung	0.80

The correlation scores and heat maps clearly demonstrate a high degree of concordance for the expression profile of this gene set derived from both platforms. Next, we compared mammary primary tumors and pulmonary metastases using the customized NanoString nCounter^**®**^ Gene Expression Codeset versus global gene expression on the Affymetrix platform. The NanoString expression counts can be found in S4 Table. The comparison of NanoString and microarray expression results for the selected NanoString gene set show similar sensitivity (S5 Table).

### Embryonic stem cell and epithelial-mesenchymal transition signature

**Fig 4:**
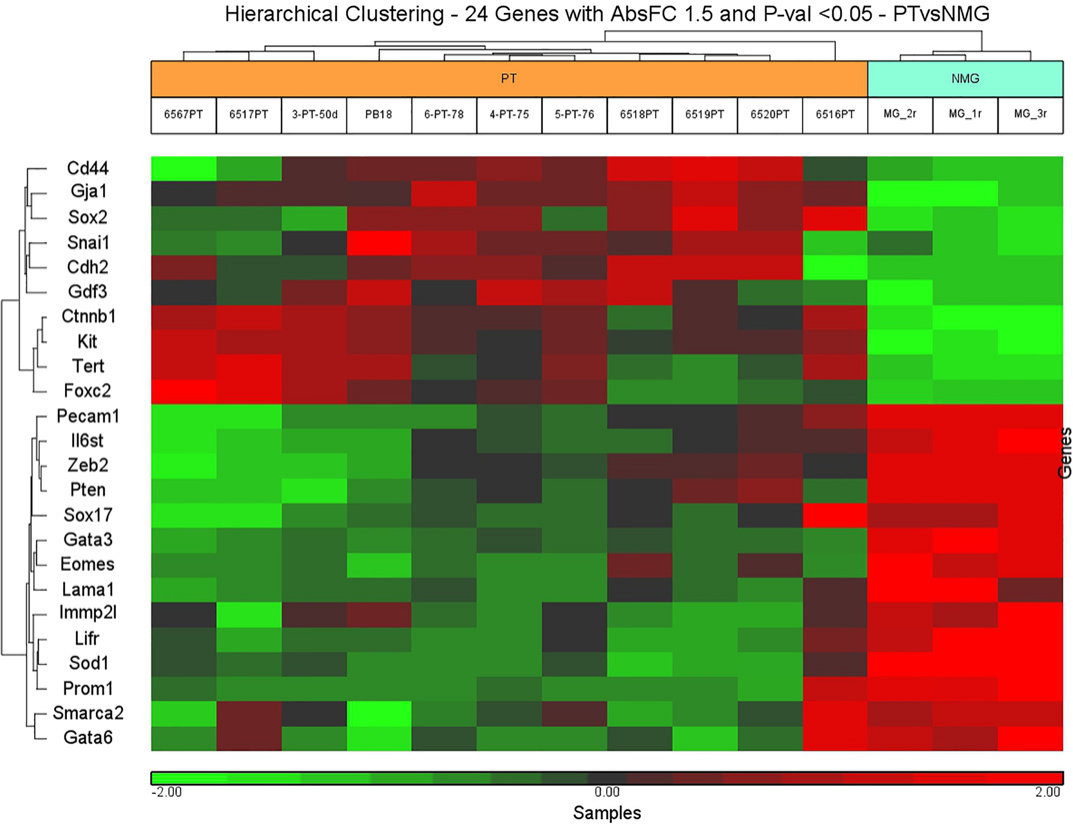
Unsupervised hierarchical clustering of normal mammary gland versus primary tumor. Scaled down representation of the entire cluster is based on 24 genes differentially expressed between normal mammary gland (NMG) and primary mammary tumors (PT). Each row represents a single gene and each column represents a sample. Red color indicates upregulation, green color—downregulation, and black color—no change in expression level compared with the reference sample.

In this study we used a customized gene set containing ESC, EMT and MET markers that were selected for nCounter® Gene Expression Assay, and the same gene set was selected from the Affymetrix platform. We conducted an analysis of variance (ANOVA) between normal mammary gland epithelium (3 samples) and primary mammary carcinoma (11 samples), identifying 24 differentially expressed genes (absolute fold change threshold of 1.5 and p-value <0.05) from the Affymetrix platform (S6 Table). The unsupervised hierarchical clustering based on the expression pattern of this gene set resulted in two main branches segregating the 2 groups of samples (Fig 4).

Overexpression of *Sox2*, *Snail*, *Cdh2*, *Ctnnb1*, *Kit*, *Tert* and *Foxc2* was observed in the majority of primary tumor samples while a distinct overexpression of *Pecam1*, *Zeb2*, *Pten*, *Gata3*, *Eomes*, *Sod1*, *Prom1* and *Gata6* was observed in normal mammary gland areas.

**Fig 5:**
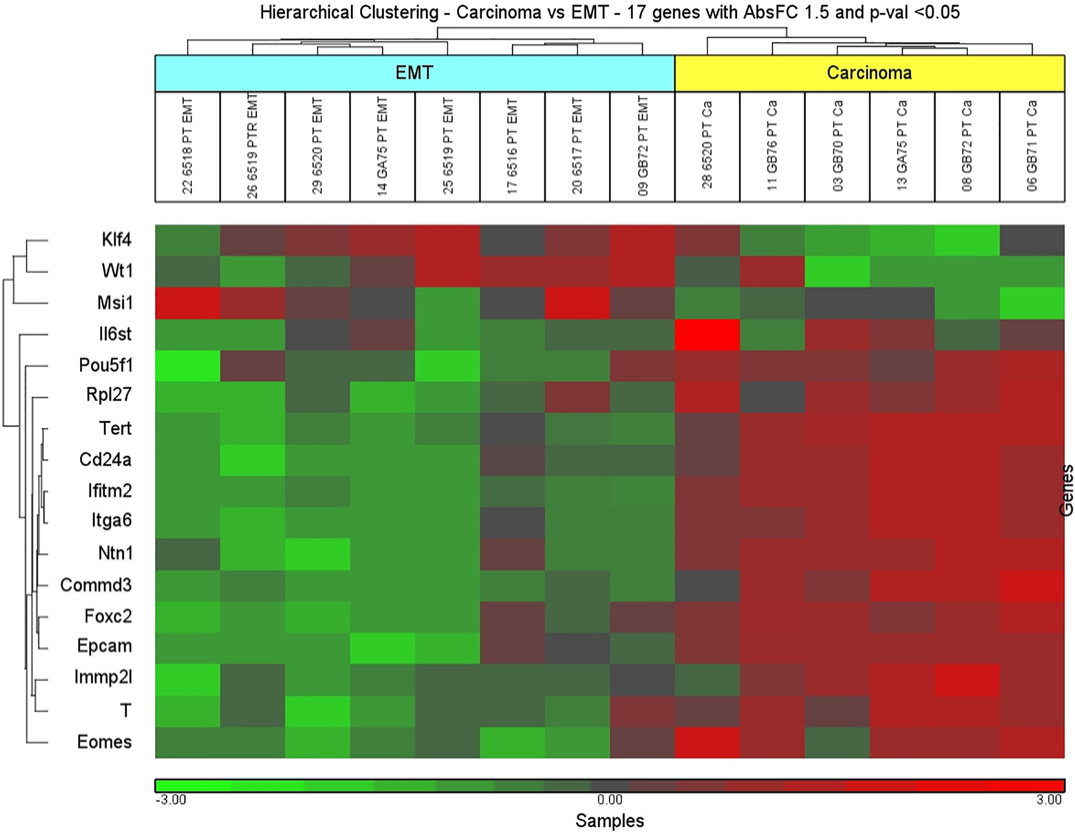
Unsupervised hierarchical clustering of carcinoma versus EMT. Scaled down representation of the entire cluster is based on 17 genes differentially expressed between carcinoma (epithelial-like areas) and EMT (spindle-like areas). Each row represents a single gene and each column represents a sample. Red color indicates upregulation, green color—downregulation, and black color—no change in expression level compared with the reference sample. EMT: Epithelial-Mesenchymal transition.

This metastatic mouse model displays a mixed cell population in the primary mammary tumors of both epithelial and spindle-like mesenchymal cells. However, in lung parenchyma only epithelial-like features were displayed. To generate a precise correlation between epithelial and spindle-like cells and their ESC and EMT-MET signature, we used LCM combined with NanoString mRNA analysis. The former procedure allowed gene expression profile analysis with a cell base, rather than tissue based resolution. To characterize molecular alterations of the ESC markers and EMT members during tumorigenesis, we conducted an ANOVA test between epithelial-like cells (6 carcinoma samples) and spindle-like (8 EMT samples) cells, identifying 17 differentially expressed genes (fold change 1.5 and p-value <0.05) (S6 Table). The unsupervised hierarchical clustering based on the expression pattern of this gene set resulted in two main branches segregating the samples in distinct groups (Fig 5).

Overexpression of *Klf4*, *Wt1* and *Msi1* was observed in the EMT (spindle-like areas) whereas overexpression of the *Epcam*, *T*, *Foxc2*, and *CD24a* was observed in carcinoma areas.

## Conclusions

Our adaptation of LCM sample preparation and workflow to the requirements of the NanoString platform allowed acquiring samples with high RNA quality. The subsequent analysis of such samples provided sensitive detection of genes of interest and their associated molecular pathways. The pilot LCM study is essential for determining the target area size containing around 100ng of RNA to avoid biased NanoString results. Through this study, we demonstrate effective coupling of LCM and NanoString technologies to achieve reliable, reproducible and robust interrogation of gene expression profiles. No amplification of mRNA is also a positive feature of the NanoString technology.

In this study we compared non-amplified LCM samples using a customized nCounter gene expression profile from NanoString technology with amplified whole tissue using a global Microarray Affymetrix profile. We observed high degree of confidence and concordance between both assays. However, the use of LCM technology focuses on precise extraction of highly defined histological areas to more fully clarify discrete molecular changes in gene expression at a finer level of resolution, followed by NanoString, which lends sensitivity to obtain expression counts without amplification, truly represents a combinatorial synergism of technologies that enables interrogation of a complex biological system with reasonable level of accuracy.

## Supporting Information

S1 Fig Demonstration of rodent sample collection for laser-capture and laser cutting microdissection. RNAse-free conditions should be observed through the entire procedure: (A) RNAse-free necropsy instruments should be stored in autoclaved packages and placed in 50 ml Falcon tubes under plastic cover (arrow) before transfer to necropsy hood; (B) Reagents and materials should be solely designated for LCM sample collection; (C) Skin (arrows) should be moved away from the peritoneal (a) and pleural (b) cavities to avoid contamination and subsequent cryosectioning artifacts from hair. To ensure prompt dissection of the target organ and to slow down RNA degradation, only the targeted area should be exposed; (D) Intact target organ (arrow) can be trimmed if required (E), and then positioned into plastic embedding mold. (F) The mold (arrow) should be completely filled with OCT and floated on a bath of dry ice and 2-methylbutane; (G) After OCT turned white, the mold should remain on a bath for 10 minutes for complete freezing of the tissue; (H) The mold with frozen tissue should be moved onto dry ice and observed for complete evaporation of 2-methylbutane prior to -80°C storage. A: Scale bar corresponds to 20 mm; B, C, F-H: Scale bars correspond to 10 mm; D: Scale bar corresponds to 2000 μm; E: Scale bar corresponds to 3000 μm.
(TIF)

S2 Fig LCM slide preparation for NanoString sample: Cryotomy of frozen OCT embedded tissue onto metal-framed PET slides for laser-cutting microdissection. (A) MMI SupportSlide with the elevated platform (black arrow) facilitates section mounting on metal-framed slide (white arrow); (B) The window of PET slide (arrow) snugly fits the elevated platform of support slide; (C) MMI SupportSlide–PET slide assembly should be flipped that inverted MMI logo of SupportSlide (arrow) faces cryotomist during mounting of the section; (D) The OCT block trimmed close to the tissue allows to fit several serial sections (arrows) in the window of PET slide; (E) Immediately after mounting, pre-labeled slides should be placed on a kimwipe in a Styrofoam box with dry ice, label down (solid arrow). Slides should be transferred in five slot pre-chilled mailing containers (double arrows) for -80°C storage prior to LCM. Scale bars correspond to 4000μm.
(TIF)

S3 Fig LCM slide preparation for NanoString sample: Demonstration of staining protocol and technique for metal-framed membrane slides. (A) LCM slides (arrow) transferred into fixative stored in a tube on dry ice inside the Styrofoam box; (B) LCM staining station: Methylgreen with RNAse inhibitor (1), 100% ethanol for brief rinse (2) prior to the application of cresyl violet/eosin stain (3), two changes of 100% ethanol (4, 5), staining box (empty pipet tip container) (6), timers (7) preset for the duration of the first and two following steps of staining protocol, kimwipes (8) for draining the slides after methylgreen and cresyl violet/eosin stains; (C) OCT removal step; (D) cresyl violet/eosin stain application; (E, F) Draining steps prior to slide transfer into 100% ethanol after Methylgreen and stain, respectively; (G, H, I, J) LCM staining protocol sequence following the fixation: OCT removal with Methylgreen, rinse in 100% ethanol, staining with cresyl violet/eosin and rinse after staining, dehydration in 100% ethanol (G), clearing in two changes of xylene (H), drying in the fume hood (I) prior to transfer into a desiccator for additional drying (J); (K) LCM slide prepared for dissection: four serial sections of tumor (arrow) are positioned on the membrane in the same orientation for time effective dissection; (L) The same target regions (arrows) are dissected and removed from the slide by MMI IsolationCap®; (M) For larger target regions, retrieval of dissected targets from the slide by forceps under the dissection microscope into 1.5 ml Eppendorf tube is more time effective than with MMI IsolationCap®. Before LCM slide removal from the dissecting stage, membrane slide should be covered with regular glass microscope slide (black arrow) to avoid the loss of cut targets (white arrows) due to static. A: Scale bar corresponds to 20 mm; B, C, E, F, I, J: Scale bars correspond to 10 mm; D, G, H, K-M: Scale bars correspond to 5 mm.
(TIF)

S4 Fig nSolver QC parameters. The table represents numerical parameters used in this study to access LCM samples quality control for NanoString gene profiling.
(TIF)

S5 Fig Agilent Bioanalyzer electropherogramm of whole tissue RNA. The view of an Agilent RNA analysis on NanoChip. RIN- RNA integrity number.
(PDF)

S6 Fig Correlation cluster image using Pearson’s Correlation statistic shows high degree of concordance between the same samples from microarray and NanoString datasets. The white squares represent high degree of confidence and concordance when comparing PT, LungMets, NMG and NL between non-amplified LCM samples using a customized nCounter gene expression profile from NanoString technology with amplified whole tissue using a global Microarray profile. PT: primary tumor; LungMets: lung metastasis; NMG: normal mammary gland and NL: normal lung parenchyma.
(TIF)

S1 Table The targeted region and corresponding sequences of embryonic stem cells and EMT-MET markers selected for a custom NanoString probe set. (XLSX)

S2 Table LCM samples collected for NanoString analysis via laser cutting and lysed in Qiagen buffer RLT (4μl of lysate contains around 100 ng of RNA). (XLSX)

S3 Table Nanostring QC parameters to all samples. (XLSX)

S4 Table NanoString expression counts. (XLSX)

S5 Table Summary of the NanoString and microarray gene expression profiling using the NanoString gene list. (XLSX)

S6 Table Data matrix with log-transformed expression intensity values of genes in PT and NMG samples (top) and Carcinoma and EMT samples (bottom), used as input for generating the heat maps in Figs 4 and 5, respectively. (XLSX)
