# Supplementary figures and images for: Adaptation of Laser Microdissection Technique for the Study of a Spontaneous Metastatic Mammary Carcinoma Mouse Model by NanoString Technologies
Source: PLoS One. 2016 Apr 14;11(4):e0153270. doi: 10.1371/journal.pone.0153270 (PMC4831786; doi:10.1371/journal.pone.0153270)

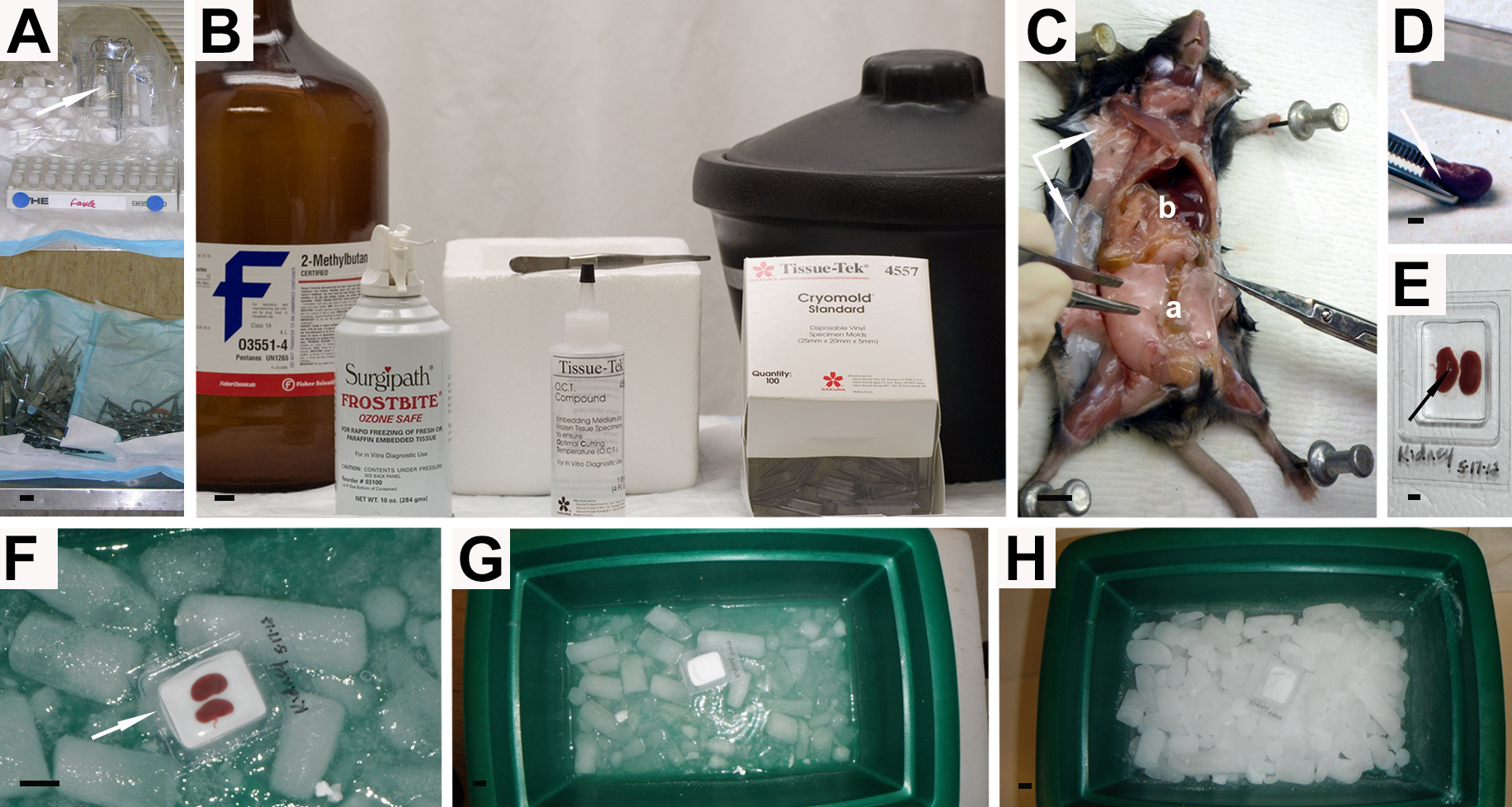

Supplement: S1 Fig — RNAse-free conditions should be observed through the entire procedure: (A) RNAse-free necropsy instruments should be stored in autoclaved packages and placed in 50 ml Falcon tubes under plastic cover (arrow) before transfer to necropsy hood; (B) Reagents and materials should be solely designated for LCM sample collection; (C) Skin (arrows) should be moved away from the peritoneal (a) and pleural (b) cavities to avoid contamination and subsequent cryosectioning artifacts from hair. To ensure prompt dissection of the target organ and to slow down RNA degradation, only the targeted area should be exposed; (D) Intact target organ (arrow) can be trimmed if required (E), and then positioned into plastic embedding mold. (F) The mold (arrow) should be completely filled with OCT and floated on a bath of dry ice and 2-methylbutane; (G) After OCT turned white, the mold should remain on a bath for 10 minutes for complete freezing of the tissue; (H) The mold with frozen tissue should be moved onto dry ice and observed for complete evaporation of 2-methylbutane prior to -80°C storage. A: Scale bar corresponds to 20 mm; B, C, F-H: Scale bars correspond to 10 mm; D: Scale bar corresponds to 2000 μm; E: Scale bar corresponds to 3000 μm. (TIF) [file pone.0153270.s001.tif]

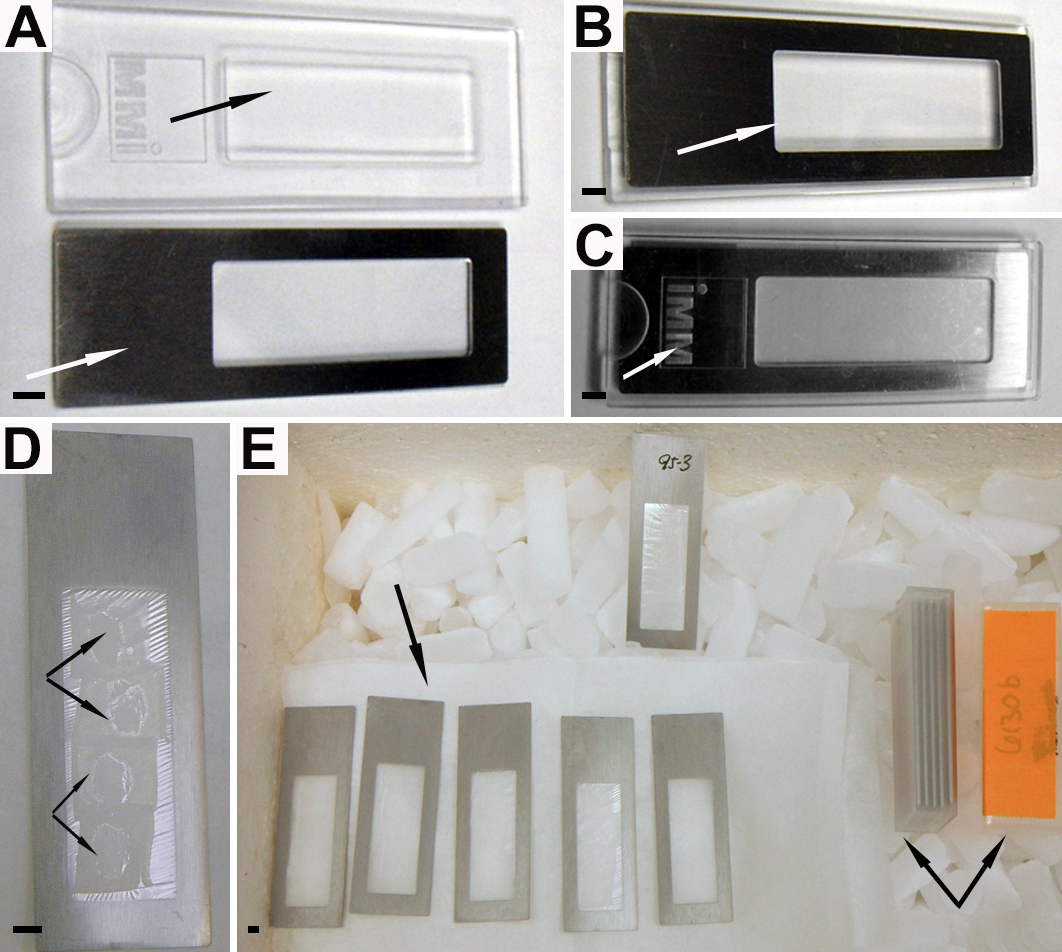

Supplement: S2 Fig — (A) MMI SupportSlide with the elevated platform (black arrow) facilitates section mounting on metal-framed slide (white arrow); (B) The window of PET slide (arrow) snugly fits the elevated platform of support slide; (C) MMI SupportSlide–PET slide assembly should be flipped that inverted MMI logo of SupportSlide (arrow) faces cryotomist during mounting of the section; (D) The OCT block trimmed close to the tissue allows to fit several serial sections (arrows) in the window of PET slide; (E) Immediately after mounting, pre-labeled slides should be placed on a kimwipe in a Styrofoam box with dry ice, label down (solid arrow). Slides should be transferred in five slot pre-chilled mailing containers (double arrows) for -80°C storage prior to LCM. Scale bars correspond to 4000μm. (TIF) [file pone.0153270.s002.tif]

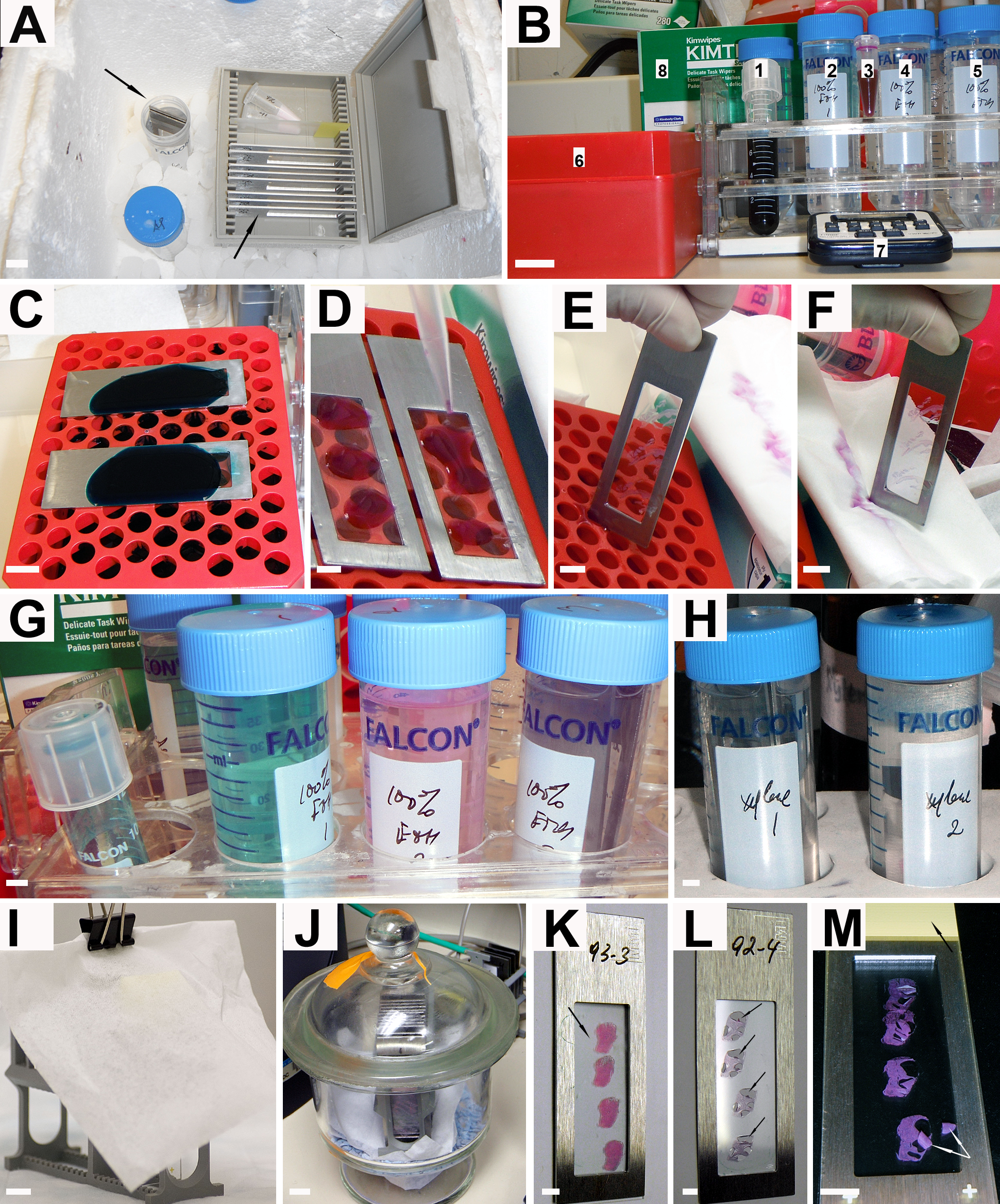

Supplement: S3 Fig — (A) LCM slides (arrow) transferred into fixative stored in a tube on dry ice inside the Styrofoam box; (B) LCM staining station: Methylgreen with RNAse inhibitor (1), 100% ethanol for brief rinse (2) prior to the application of cresyl violet/eosin stain (3), two changes of 100% ethanol (4, 5), staining box (empty pipet tip container) (6), timers (7) preset for the duration of the first and two following steps of staining protocol, kimwipes (8) for draining the slides after methylgreen and cresyl violet/eosin stains; (C) OCT removal step; (D) cresyl violet/eosin stain application; (E, F) Draining steps prior to slide transfer into 100% ethanol after Methylgreen and stain, respectively; (G, H, I, J) LCM staining protocol sequence following the fixation: OCT removal with Methylgreen, rinse in 100% ethanol, staining with cresyl violet/eosin and rinse after staining, dehydration in 100% ethanol (G), clearing in two changes of xylene (H), drying in the fume hood (I) prior to transfer into a desiccator for additional drying (J); (K) LCM slide prepared for dissection: four serial sections of tumor (arrow) are positioned on the membrane in the same orientation for time effective dissection; (L) The same target regions (arrows) are dissected and removed from the slide by MMI IsolationCap®; (M) For larger target regions, retrieval of dissected targets from the slide by forceps under the dissection microscope into 1.5 ml Eppendorf tube is more time effective than with MMI IsolationCap®. Before LCM slide removal from the dissecting stage, membrane slide should be covered with regular glass microscope slide (black arrow) to avoid the loss of cut targets (white arrows) due to static. A: Scale bar corresponds to 20 mm; B, C, E, F, I, J: Scale bars correspond to 10 mm; D, G, H, K-M: Scale bars correspond to 5 mm. (TIF) [file pone.0153270.s003.tif]

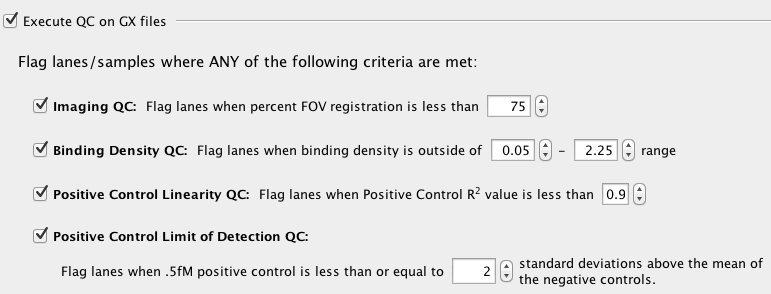

Supplement: S4 Fig — The table represents numerical parameters used in this study to access LCM samples quality control for NanoString gene profiling. (TIF) [file pone.0153270.s004.tif]

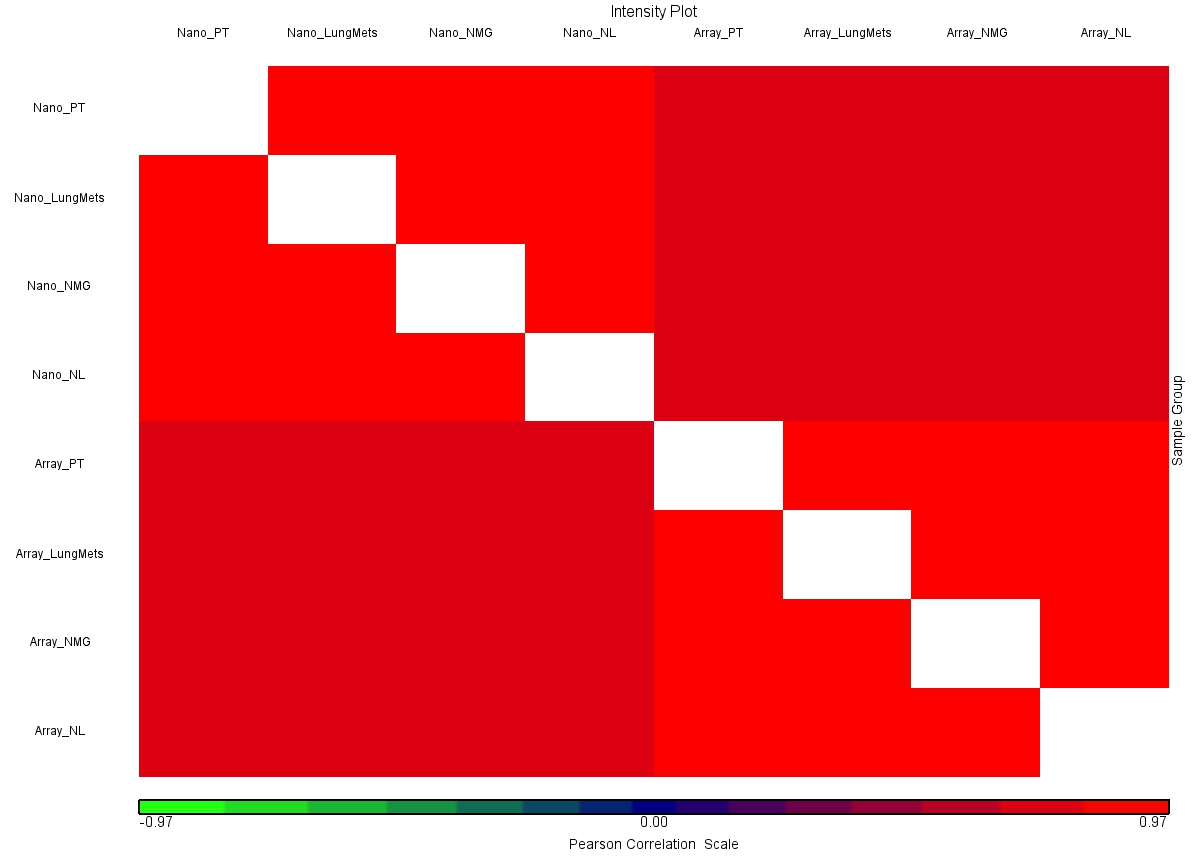

Supplement: S6 Fig — The white squares represent high degree of confidence and concordance when comparing PT, LungMets, NMG and NL between non-amplified LCM samples using a customized nCounter gene expression profile from NanoString technology with amplified whole tissue using a global Microarray profile. PT: primary tumor; LungMets: lung metastasis; NMG: normal mammary gland and NL: normal lung parenchyma. (TIF) [file pone.0153270.s006.tif]
