# Supplementary material for: Adaptation of Laser Microdissection Technique for the Study of a Spontaneous Metastatic Mammary Carcinoma Mouse Model by NanoString Technologies
Source: PLoS One. 2016 Apr 14;11(4):e0153270. doi: 10.1371/journal.pone.0153270 (PMC4831786; doi:10.1371/journal.pone.0153270)
Supplement: S5 Fig — The view of an Agilent RNA analysis on NanoChip. RIN- RNA integrity number. (PDF) [file pone.0153270.s005.pdf]

Assay Class: Eukaryote Total RNA Nano
Data Path: V:\...\CSAS_14023_Castro_011513\CSAS_14023_Castro_QC_1_011513.xad

Created: 1/15/2013 1:31:48 PM
Modified: 1/15/2013 2:03:03 PM

Electrophoresis File Run Summary

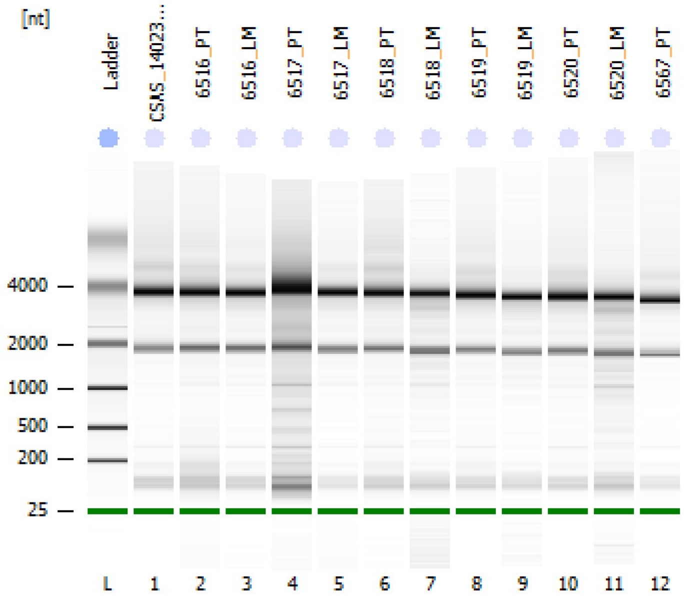

Instrument Information:

Instrument Name: DE13804808
Firmware: C.01.069
Serial#: DE13804808
Type: G2939A

Assay Information:

Assay Origin Path: C:\Program Files\Agilent\2100 bioanalyzer\2100 expert\assays\RNA\Eukaryote Total RNA Nano Series II.xsy

Assay Class: Eukaryote Total RNA Nano

Version: 2.6

Assay Comments: Total RNA Analysis ng sensitivity (Eukaryote)

© Copyright 2003 - 2009 Agilent Technologies, Inc.

Chip Information:

Chip Lot #:

Reagent Kit Lot #:

Chip Comments:

CSAS_14023_Castro_PB18_PT

RIN:10

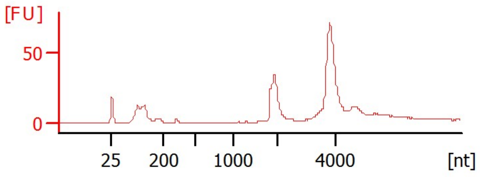

6516_PT

RIN: 9.60

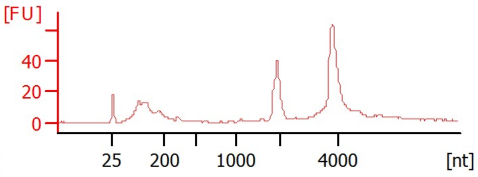

6516_LM

RIN:10

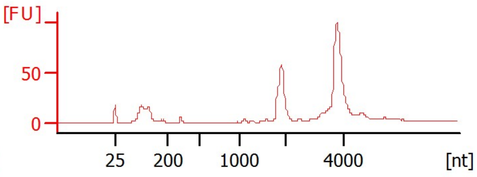

6517_PT

RIN: 8.40

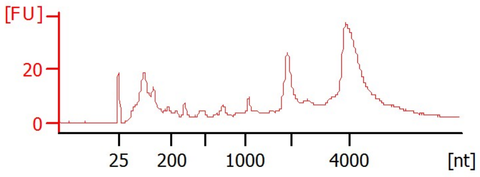

6517_LM

RIN:10

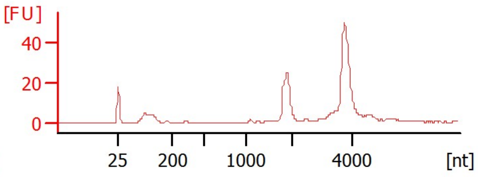

6518_PT

RIN:10

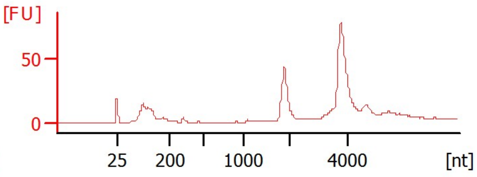

6518_LM

RIN: 9.90

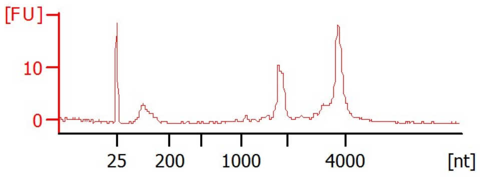

6519_PT

RIN: 9.80

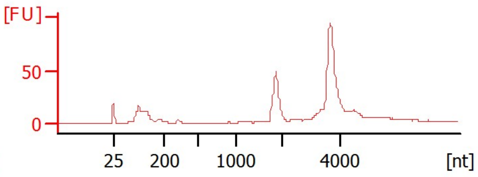

6519_LM

RIN: 9.90

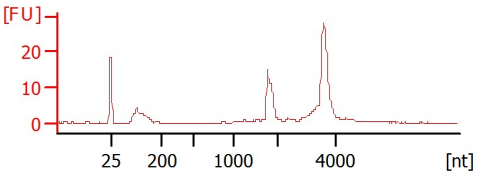

6520_PT

RIN: 9.70

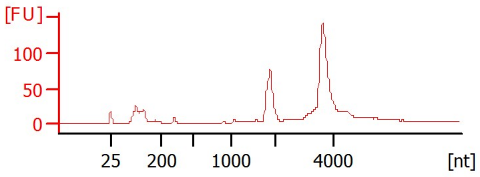

6520_LM

RIN: 8.30

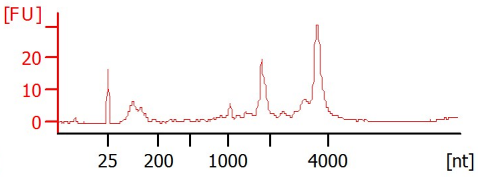

6567_PT

RIN:10

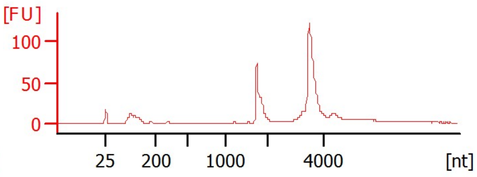

© Copyright 2003 - 2009 Agilent Technologies, Inc.
